# Effectiveness of Whole-Virus COVID-19 Vaccine among Healthcare Personnel, Lima, Peru

**DOI:** 10.3201/eid2813.212477

**Published:** 2022-12

**Authors:** Carmen S. Arriola, Giselle Soto, Matthew Westercamp, Susan Bollinger, Angelica Espinoza, Max Grogl, Alejandro Llanos-Cuentas, Eduardo Matos, Candice Romero, Maria Silva, Rachel Smith, Natalie Olson, Michael Prouty, Eduardo Azziz-Baumgartner, Fernanda C. Lessa

**Affiliations:** Centers for Disease Control and Prevention, Atlanta, Georgia, USA (C.S. Arriola, M. Westercamp, S. Bollinger, R. Smith, N. Olson, E. Azziz-Baumgartner, F.C. Lessa);; Naval Medical Research Unit No. 6, Lima, Peru (G. Soto, A. Espinoza, M. Grogl, C. Romero, M. Silva, M. Prouty);; Cayetano Heredia Hospital, Lima (A. Llanos-Cuentas);; Arzobispo Loayza National Hospital, Lima (E. Matos)

**Keywords:** COVID-19, respiratory infections, severe acute respiratory syndrome coronavirus 2, SARS-CoV-2, SARS, coronavirus disease, zoonoses, viruses, coronavirus, vaccines, Peru

## Abstract

In February 2021, Peru launched a COVID-19 vaccination campaign among healthcare personnel using an inactivated whole-virus vaccine. The manufacturer recommended 2 vaccine doses 21 days apart. We evaluated vaccine effectiveness among an existing multiyear influenza vaccine cohort at 2 hospitals in Lima. We analyzed data on 290 participants followed during February–May 2021. Participants completed a baseline questionnaire and provided weekly self-collected nasal swab samples; samples were tested by real-time reverse transcription PCR. Median participant follow-up was 2 (range 1–11) weeks. We performed multivariable logistic regression and adjusted for preselected characteristics. During the study, 25 (9%) participants tested SARS-CoV-2–positive. We estimated adjusted vaccine effectiveness at 95% (95% CI 70%–99%) among fully vaccinated participants and 100% (95% CI 88%–100%) among partially vaccinated participants. These data can inform the use and acceptance of inactivated whole-virus vaccine and support vaccination efforts in the region.

Peru is a middle-income country disproportionately affected by COVID-19 and struggling to protect its essential workforce (*1*–*4*). Despite early lockdowns, curfews, and other public health and social measures implemented to reduce disease spread (*5*), by May 22, 2021, Peru had 180,764 reported COVID-19–associated deaths and continued to accrue cases (*6*,*7*). As in many other middle-income countries, healthcare services in Peru were overwhelmed with patients, had limited personal protective equipment, and had delayed and limited COVID-19 vaccination, leading to unrest and strikes among healthcare personnel (*8*). On February 9, 2021, Peru initiated COVID-19 vaccination with the Beijing Institute of Biologic Products Coronavirus Vaccine (BBIBP-CorV; Sinopharm, https://www.sinopharm.com), an inactivated whole-virus vaccine. Healthcare personnel were a priority group for vaccination. During the study period (February 9–May 4, 2021), BBIBP-CorV vaccine was the only COVID-19 vaccine available for healthcare personnel in Peru (*9*,*10*). The manufacturer recommended 2 vaccine doses 21 days apart. 

Evidence on BBIBP-CorV vaccine effectiveness could reduce hesitancy about the vaccine and support vaccination efforts. We used an existing multiyear influenza vaccine cohort of healthcare workers at 2 hospitals in Lima (*11*) to evaluate BBIBP-CorV vaccine effectiveness at preventing symptomatic and asymptomatic SARS-CoV-2 infections.

## Methods

### Study Design and Population

We designed a prospective cohort study that we conducted at 2 tertiary hospitals in Lima, Peru, during February 9–May 4, 2021. We invited healthcare workers 18–65 years of age from both hospitals to participate in the cohort. For study inclusion, participants had to work full-time (>30 hours per week) at the facility; have routine, direct, hands-on or face-to-face contact with patients (within 1 m) as part of a typical work shift; and have worked at the facility for >1 year before enrollment.

### Data Collection

Participants provided written informed consent and completed a baseline questionnaire about their demographic characteristics and role in the hospital. Questions included information on self-reported exposure to COVID-19 patients, work in the intensive care unit (ICU), or work in the emergency department (ED). Participants provided serum samples at baseline and at the end of the study period. Each participant was followed for up to 16 weeks after enrollment. Participants responded to a weekly survey that included questions about COVID-19 exposure and receipt of BBIBP-CorV vaccine as documented by the hospitals. Participants also provided a weekly self-collected anterior nasal swab sample, which was tested for SARS-CoV-2 by real-time reverse transcription PCR (rRT-PCR) at the US Naval Medical Research Unit 6 (NAMRU-6) in Lima, following testing protocols from the US Centers for Disease Control and Prevention (CDC) (*12*). rRT-PCR testing was performed in pools of 5 samples; if pools tested positive, all 5 individual samples were tested separately. Serum samples were shipped to CDC (Atlanta, Georgia, USA) for pan-Ig serologic testing (B. Freeman et al., unpub. data, https://doi.org/10.1101/2020.04.24.057323).

We considered participants fully vaccinated starting 14 days after receipt of their second dose and partially vaccinated starting 14 days after receipt of the first dose and participants not meeting these criteria as unvaccinated. This study was reviewed and approved by the NAMRU-6 institutional review board. 

### Statistical Analysis

We compiled healthcare personnel demographics, occupational information, baseline serology, COVID-19 vaccine receipt, and laboratory detection of SARS-CoV-2. We applied χ^2^ or Wilcoxon tests, as appropriate, to assess differences in demographics, occupational information, and baseline serology, stratified by SARS-CoV-2 detection and COVID-19 vaccine receipt.

We estimated vaccine effectiveness by using a multivariable logistic regression model adjusted for preselected characteristics, including age, sex, exposure to COVID-19 patients, work in ICU or ED, body mass index (BMI), and time of follow-up in days. We defined vaccine effectiveness as [1 – adjusted odds ratio] × 100% and calculated 95% CIs. For these analyses, we excluded persons who were seropositive at baseline and those with a positive COVID-19 test before February 9, 2021. The partial vaccination model only included participants who received 1 dose of the vaccine during the study period. Partially vaccinated participants were excluded from the full vaccination analysis. We calculated COVID-19 vaccine effectiveness under both full and partial vaccination scenarios. The outcome of interest in the model was SARS-CoV-2 detection; if SARS-CoV-2 was detected in a participant before first vaccination date or before the 2-week period after first vaccination, we considered the participant unvaccinated for the analysis. We conducted all analyses in R version 4.1.0 (R Foundation for Statistical Computing, https://www.r-project.org).

## Results

### Study Sample Characteristics, SARS-CoV-2 Infections, and COVID-19 Vaccine Receipt

The participant cohort comprised 290 healthcare workers followed during February 9–May 4, 2021; a total of 270 (93.1%) participants reported receiving >1 COVID-19 vaccine dose, 80% (216/270) of whom reported being fully vaccinated before the end of the follow-up period. The median follow-up period was 2 (range 1–11) weeks after the 2-week postvaccination period. Median age of participants was 45 (interquartile range [IQR] 38–52) years. Among all participants, 74% (215/290) were female, and 90% (260/290) reported being of mixed race. Only 3% (8/290) of participants reported a chronic medical condition, including asthma, diabetes, high blood pressure, chronic heart disease, autoimmune condition, HIV/AIDS, or other medical conditions requiring clinical care for >6 months. Among participants, 49% (143/290) were classified as overweight (BMI 25 to <30) and 22% (64/290) as obese (BMI >30). Over one third (106/290) of participants had a reactive result for SARS-CoV-2 pan-Ig antibodies on baseline serum samples, and SARS-CoV-2 was detected by rRT-PCR among 25 (9%) participants during follow-up. Participants who were seronegative at baseline were more likely to subsequently test positive for SARS-CoV-2 through rRT-PCR than participants who were seropositive at baseline (p<0.001) (Table 1).

**Table 1:** Characteristics, vaccine receipt, and SARS-CoV-2 laboratory detection among 290 participants in a study on effectiveness of whole-virus COVID-19 vaccine among healthcare personnel, Lima, Peru, February 9–May 4, 2021*

Characteristics	All workers	SARS-CoV-2 testing†				Vaccination status‡				
		Positive	Negative	p value		Unvaccinated	Partially vaccinated	p value	Fully vaccinated	p value
Total no. (%)	290 (100)	25 (9)	265 (91)	NA		20 (7)	54 (19)	NA	216 (74)	NA
Median age, y (IQR)	45 (38–52)	48 (41–54)	45 (38–51)	0.82		39 (37–49)	47 (39–52)	0.14	45 (39–52)	0.12
Age range, y										
18–39	85 (29)	6 (24)	79 (30)	NA		10 (50)	14 (26)	NA	61 (28)	NA
40–49	110 (38)	10 (40)	100 (38)	NA		5 (25)	22 (41)	NA	83 (38)	NA
50–65	95 (33)	9 (36)	86 (32)	NA		5 (25)	18 (33)	NA	72 (33)	NA
Sex										
M	75 (26)	6 (24)	69 (26)	1.0		1 (5)	25 (46)	<0.01	49 (23)	0.12
F	215 (74)	10 (76)	196 (74)	NA		19 (95)	29 (54)	NA	167 (77)	NA
Race/ethnicity										
Mixed race	260 (90)	21 (84)	239 (90)	0.45		19 (95)	45 (83)	0.48	196 (91)	0.89
Indigenous	19 (7)	2 (8)	17 (6)	NA		1 (5)	3 (6)	NA	15 (7)	NA
Black	8 (3)	1 (4)	7 (3)	NA		0	5 (9)	NA	3 (1)	NA
White	3 (1)	1 (4)	2 (1)	NA		0	1 (2)	NA	2 (1)	NA
Education										
High school only	37 (13)	2 (8)	35 (13)	0.75		0	13 (24)	0.02	24 (11)	0.10
Associate or bachelor’s degree	233 (80)	21 (84)	212 (80)	NA		20 (100)	38 (70)	NA	175 (81)	NA
Postgraduate education	20 (7)	2 (8)	18 (7)	NA		0	3 (6)	NA	17 (8)	NA
Comorbidities										
Any medical condition§	8 (3)	2 (8)	6 (2)	0.52		1 (5)	2 (4)	0.74	5 (2)	0.64
BMI¶										
Normal	83 (29)	9 (36)	74 (28)	0.61		9 (45)	17 (31)	0.47	57 (26)	0.20
Overweight	143 (49)	12 (48)	131 (49)	NA		7 (35)	27 (50)	NA	109 (50)	NA
Obese	64 (22)	4 (16)	60 (23)	NA		4 (20)	10 (19)	NA	50 (23)	NA
Smoking daily/some	11 (4)	1 (4)	10 (4)	1.0		1 (5)	3 (6)	1.0	7 (3)	1.0
Job type										
Physician	11 (4)	1 (4)	10 (4)	0.62		0	0	<0.01	11 (5)	0.08
Nurse	63 (22)	2 (8)	61 (23)	NA		1 (5)	10 (19)	NA	52 (24)	NA
Midwife or dentist	12 (4)	1 (4)	11 (4)	NA		0	0	NA	12 (5)	NA
Technician, assistant	135 (47)	14 (56)	121 (46)	NA		15 (75)	17 (31)	NA	103 (48)	NA
Pharmacist, social worker, nutritionist	2 (1)	0	2 (1)	NA		1 (5)	0	NA	1 (0)	NA
Physical therapist	4 (1)	0	4 (2)	NA		0	2 (4)	NA	2 (1)	NA
Administrator, security, maintenance, transporter	49 (17)	7 (28)	42 (15)	NA		1 (5)	20 (37)	NA	28 (12)	NA
Other	14 (5)	0	14 (5)	NA		2 (10)	5 (9)	NA	7 (3)	NA
Exposed to COVID-19 patients in healthcare setting	249 (86)	18 (72)	231 (87)	0.59		17 (85)	45 (83)	1.0	187 (87)	1.0
ICU	27 (9)	4 (16)	23 (9)	0.40		0	8 (15)	0.16	19 (9)	0.34
ED	101 (35)	11 (44)	90 (34)	0.43		9 (45)	20 (37)	0.72	72 (33)	0.42
Median hours worked at site/week (IQR)	36 (36–36)	36 (36–40)	36 (36–36)	0.93		36 (36–39)	36 (36–48)	0.23	36 (36–36)	0.40
Median hours patient-provider face-to-face/week (IQR)	30 (24–36)	30 (20–30)	30 (25–36)	0.04		33 (30–37)	30 (25–36)	0.31	30 (24–36)	0.11
Reactive SARS-CoV-2 serology at baseline	106 (37)	0	106 (40)	<0.01		5 (25)	15 (28)	1.0	86 (40)	0.28

*Values represent no. (%) unless otherwise indicated. p values were calculated by using χ^2^ test for categorical and Wilcoxon signed rank test for continuous variables. BMI, body mass index; ED, emergency department; ICU, intensive care unit. †At least once by weekly testing during follow-up period. ‡Vaccination of healthcare workers started in Lima on February 9, 2021, and was assessed by interview on a weekly basis. Partially vaccinated refers to persons who received 1 dose of whole-virus COVID-19 vaccine during the study period; fully vaccinated refers to persons who received 2 doses of whole-virus COVID-19 vaccine during the study period. Partially and fully vaccinated groups were separately compared against unvaccinated persons. §Asthma, diabetes, high blood pressure, chronic heart disease, autoimmune condition, HIV/AIDS, another medical condition requiring clinical care >6 mo. ¶Normal (18.5 to <25); overweight (25 to <30); obese (>30).

### COVID-19 Vaccine Effectiveness

After excluding participants who were seropositive at baseline and those with a positive COVID-19 test before February 9, 2021, and adjusting for age, sex, exposure to COVID-19 patients, work in the ICU, work in the ED, BMI, and time of follow-up in days, we estimated overall BBIBP-CorV vaccine effectiveness against symptomatic or asymptomatic SARS-CoV-2 infection as 97% (95% CI 88%–99%) for those who received >1 dose of the vaccine. Effectiveness was 100% (95% CI 88%–100%) for partially vaccinated participants and 95% (95% CI 70%–99%) for fully vaccinated participants (Table 2).

**Table 2:** COVID-19 vaccine effectiveness by number of doses received in a study on effectiveness of whole-virus COVID-19 vaccine among healthcare personnel, Lima, Peru, February 9–May 4, 2021*

Vaccination status†	COVID-19 cases			Non—COVID-19 cases			Vaccine effectiveness, % (95% CI)‡	
Vaccinated	Unvaccinated	Vaccinated	Unvaccinated	Unadjusted	Adjusted
Received >1 vaccine dose	10	9		138	6		95 (84–99)	97 (88–99)
Fully vaccinated	5	9		36	6		91 (63–98)	95 (70–99)
Partially vaccinated	5	9		25	6		87 (45–97)	100 (88–100)

*Totals exclude persons with reactive SARS-CoV-2 serology (n = 106) and persons with positive COVID-19 test before February 9, 2021 (n = 17). †Persons who tested positive before vaccination date or before the 2-week period after vaccination were considered unvaccinated for the model. We defined full vaccination as the period starting 14 d after receipt of the second dose and partial vaccination as the period starting 14 d after receipt of the first dose. Participants not meeting these criteria were considered unvaccinated. The partial vaccination model only included persons who received 1 dose of the vaccine during the study period. ‡Adjusted for age, sex, exposure to COVID-19 patients, work in the intensive care unit, work in the emergency department, body-mass index, and time of follow-up in days.

## Discussion

Among vaccinated participants in this cohort, we estimate BBIBP-CorV vaccine was >90% effective in preventing SARS-CoV-2 infection in the weeks immediately after vaccination. Furthermore, our findings indicate that, during February–May 2021, 1 of 10 study participants in 2 tertiary hospitals in Lima were infected with laboratory-confirmed SARS-CoV-2.

Healthcare personnel are at increased risk for SARS-CoV-2 infection (*13*). Our findings show continued detection of SARS-CoV-2 infection during the study period. In Peru, estimates reported >600 physicians and nurses had died of COVID-19 by June 2021 (*1*). Protecting the healthcare workforce is a global priority to ensure healthcare delivery to the population. The World Health Organization (WHO) Strategic Advisory Group of Experts on Immunization roadmap for prioritizing use of COVID-19 vaccines in the context of limited supplies includes healthcare personnel as one of the highest priority groups for vaccination (*14*). The government of Peru initiated COVID-19 vaccination on February 9, 2021, and healthcare personnel were the initial targeted group to receive the vaccine (*15*).

Our study indicates the BBIBP-CorV vaccine is effective against SARS-CoV-2 infection in the period immediately after vaccination. Our findings are compatible with those reported by WHO, in which BBIBP-CorV vaccine efficacy was estimated at 78.9% (95% CI 65.8%–87%) against COVID-19 disease in an unpublished clinical trial, with a follow-up time of 2 months (*16*). Furthermore, our findings are consistent with interim estimates published by WHO, in which vaccine effectiveness against rRT-PCR–confirmed cases among adults >18 years of age in Bahrain was 90% (95% CI 88%–91%) (*17*).

In our study, we suspect that B.1.1.1 (Alpha) was the dominant circulating SARS-CoV-2 variant in early 2021 because it was detected in 43% (n = 23) of the samples that were sequenced. However, SARS-CoV-2 variant P.1 (Gamma) was identified in Peru in January 2021 (*18*); in addition, P.1 was identified in one of the 19 samples collected during January–February 2021 (data not shown). P.1 emerged in Brazil in mid-November 2020 and rapidly spread in the state of Amazonas in early 2021, causing several hospitalizations and deaths (*19*,*20*). WHO included P.1 as a variant of concern in January 2021 because of its increased transmissibility and virulence (*21*). Data collection over time are needed to assess vaccine effectiveness under real-life circumstances as new variants emerge and circulate.

Because of high COVID-19 illness and death rates, BBIBP-CorV vaccine was rolled out in Peru and numerous other countries despite the lack of robust effectiveness data (*22*). Long-term effectiveness data are still needed, but the results from our study support continued use of BBIBP-CorV, at least in the absence of available vaccines with proven long-term effectiveness. Data from this study can be used to support vaccination in the region because offering vaccine effectiveness data can improve vaccine uptake (*23*). Unlike some other COVID-19 vaccines, BBIBP-CorV does not require complicated cold chain logistics, such as ultralow freezer conditions, and can be used within the existing cold chain infrastructure of other national immunization programs (*24*).

Among our study’s strengths is that we were able to rapidly implement a prospective cohort study by leveraging an ongoing prospective cohort established to evaluate influenza vaccine effectiveness among healthcare personnel with weekly nasal swab sampling and testing for SARS-CoV-2, regardless of symptoms. The frequency and breadth of sampling among our cohort enabled greater detection of infection than passive surveillance systems. Participation rate in this COVID-19 study was high (85%) and remained high throughout the 16-week follow-up period; >96% of participants submitted swab specimens in >13 of the 16 weeks of follow-up. SARS-CoV-2 infection was confirmed through rRT-PCR in NAMRU-6’s high proficiency laboratory, following CDC’s SARS-CoV-2 diagnostic protocol, and did not rely on point of care testing with less sensitive assays.

The first limitation of our study is that the high vaccine effectiveness we observed might be related to the short follow-up period after vaccination, 1–11 (median 2) weeks after the 2-week postvaccination period; a longer follow-up period is necessary to fully evaluate the long-term effectiveness of the vaccine among this study population. Second, we did not estimate sample size for this study to measure vaccine effectiveness so that maximum sample could be achieved; the resulting sample size was insufficient to stratify vaccine effectiveness estimates by variant or by symptomatic versus asymptomatic infection. Third, because of the limited availability of laboratory staff and high volume of weekly respiratory specimens, we implemented a pooling strategy for SARS-CoV-2 testing, which might have decreased sensitivity to detect participants with low viral shedding. Finally, our study could not distinguish nasal carriage of the virus from lower respiratory tract SARS-CoV-2 infection.

In summary, 1 in 10 healthcare personnel in our study in Peru tested positive for SARS-CoV-2 during February–May 2021. Vaccination of healthcare personnel with BBIBP-CorV vaccine was effective at reducing SARS-CoV-2 infections in the weeks immediately after vaccination. Our data support Peru’s ongoing COVID-19 vaccination efforts for reducing SARS-CoV-2 infections, especially among this critical workforce of healthcare professionals.
